# Experimental Investigation of Phase Equilibria in the Co-Ta-Si Ternary System

**DOI:** 10.3390/ma15093097

**Published:** 2022-04-25

**Authors:** Cuiping Wang, Xiang Huang, Liangfeng Huang, Mujin Yang, Peng Yang, Yunrui Cui, Jinbin Zhang, Shuiyuan Yang, Xingjun Liu

**Affiliations:** 1 College of Materials and Fujian Provincial Key Laboratory of Materials Genome, Xiamen University, Xiamen 361005, China; wangcp@xmu.edu.cn (C.W.); hx850024430@163.com (X.H.); lfhuang123@163.com (L.H.); 20720191150042@stu.xmu.edu.cn (P.Y.); 13666090301@139.com (Y.C.); jbzhang@xmu.edu.cn (J.Z.); yangshuiyuan@xmu.edu.cn (S.Y.); 2 Xiamen Key Laboratory of High Performance Metals and Materials, Xiamen University, Xiamen 361005, China; 3 Department of Mechanical and Energy Engineering, Southern University of Science and Technology, Shenzhen 518055, China; 4 School of Materials Science and Engineering, Institute of Materials Genome & Big Data, Harbin Institute of Technology, Shenzhen 518055, China

**Keywords:** Co-based superalloy, phase equilibria, Co-Ta-Si ternary system

## Abstract

In this work, two isothermal sections of the Co-Ta-Si ternary system at 900 °C and 1100 °C are constructed in the whole composition range via phase equilibrium determination with the help of electron probe microanalysis (EPMA) and X-ray diffraction (XRD) techniques. Firstly, several reported ternary phases G (Co_16_Ta_6_Si_7_), G″ (Co_4_TaSi_3_), E (CoTaSi), L (Co_3_Ta_2_Si) and V (Co_4_Ta_4_Si_7_) are all re-confirmed again. The G″ phase is found to be a kind of high-temperature compound, which is unstable at less than 1100 °C. Additionally, the L phase with a large composition range (Co_32–62_Ta_26–36_Si_10–30_) crystallizes with a hexagonal crystal structure (space group: P6_3_/*mmc*, C14), which is the same as that of the binary high-temperature λ_1_-Co_2_Ta phase. It can be reasonably speculated that the ternary L phase results from the stabilization toward low-temperature of the binary λ_1_-Co_2_Ta through adding Si. Secondly, the binary CoTa_2_ and SiTa_2_ phases are found to form a continuous solid solution phase (Co, Si)Ta_2_ with a body-centered tetragonal structure. Thirdly, the elemental Si shows a large solid solubility for Co-Ta binary compounds while the Ta and Co are hardly dissolved in Co-Si and Ta-Si binary phases, respectively.

## 1. Introduction

The γ′-Co_3_(Al, W) phase was found to be highly coherent orientation with the γ-Co matrix in the Co-based superalloys similar to the Ni-based superalloys, thus making Co-based superalloys expected to become the next generation of high-temperature structural materials such as aero-engine blades and turbine disk [1,2]. However, the novel Co-based superalloys have severe problems, such as high density and poor stability of γ′ phase at high temperatures, which limit their further development [3,4,5]. Previous studies have shown that the addition of alloying elements such as Ni, Si, Cr, V, Ta, Nb, and Ru can effectively improve the above-mentioned problems and enhance the overall mechanical properties [6,7,8,9,10,11,12]. The addition of Ta can improve the stability, volume fraction and solution temperature of the γ′ phase, and increase the stress required for the internal slip of the γ′ phase to improve the high-temperature mechanical properties of the cobalt-based superalloys [6,9,12]. The addition of Si can improve the oxidation resistance and reduce the density of the Co-based superalloys while maintaining the stability of γ′-phase and high-temperature mechanical properties of the superalloys [10,11]. However, interactions between alloying elements may also cause negative effects. For instance, the excessive addition of Si and Ta in Co-based superalloys promotes the formation of detrimental topological close-packed (TCP) phases that reduce the strength and ductility [13]. Therefore, theoretical and experimental research on the sub-systems of Co-based superalloy is needed to understand the interrelationship between composition and crystal structure, the related work has achieved considerable achievements [14,15,16,17,18,19,20,21]. Among them, the phase diagram is the theoretical fundamental to guiding the composition design and microstructure control of Co-based superalloys [22,23]. In order to better understand the relationship between the composition and microstructure of the critical Co-based superalloy system Co-Ni-Al-W-Ta-Ti-Hf-Cr-Si [24,25], the phase equilibria of the related sub-system Co-Ta-Si is of great importance.

**Figure 1:**
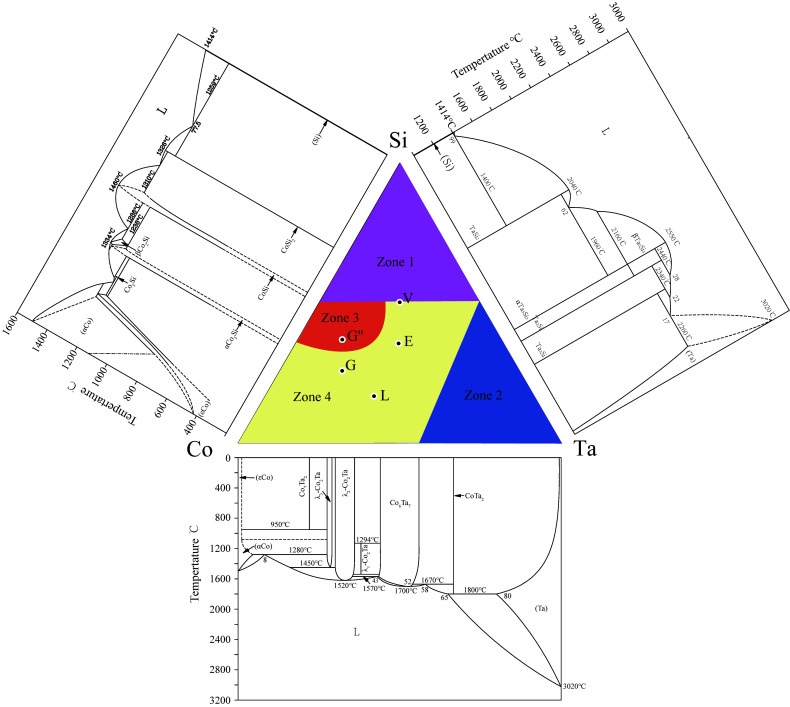
The sub-binary phase diagrams of Co-Si [26], Co-Ta [27] and Ta-Si [28] systems.

The Co-Ta-Si ternary system is constituted by three sub-binary systems: Co-Si [26], Co-Ta [27] and Ta-Si [28], as shown in Figure 1. The crystal structure information of each equilibrium phases in the Co-Ta-Si ternary system and three sub-binary systems are shown in Table 1. Firstly, there are five intermediate phases in the Co-Si binary system, namely Co_3_Si, αCo_2_Si, βCo_2_Si, CoSi and CoSi_2_ and six intermediate phases in the Co-Ta binary system: Co_7_Ta_2_, Co_6_Ta_7_, CoTa_2_, λ_1_-Co_2_Ta, λ_2_-Co_2_Ta and λ_3_-Co_2_Ta. Among them, the λ_1_-Co_2_Ta is a high-temperature MnZn_2_-type Laves phase, which decomposes into the CoTa and λ_2_-Co_2_Ta phases by an eutectic reaction at 1294 °C. Additionally, the λ_2_-Co_2_Ta and λ_3_-Co_2_Ta phases are the MnCu_2_- and MgNi_2_-type Laves phases, respectively. The CoTa_2_ Laves phase is a kind of Al_2_Cu-type, which is the same as that of the Ta_2_Si. Besides, there are six intermediate compounds in the Ta-Si binary system, namely Ta_3_Si, Ta_2_Si, αTa_5_Si_3_, βTa_5_Si_3_, γTa_5_Si_3_, and TaSi_2_. The conversion among αTa_5_Si_3_, βTa_5_Si_3_ and γTa_5_Si_3_ are completed through crystal transformation.

Previous studies have reported five ternary compounds in the Co-Ta-Si ternary system: G (Co_16_Ta_6_Si_7_) [29], G″ (Co_4_TaSi_3_) [30,31], L (Co_3_Ta_2_Si) [32,33], E (CoTaSi) [29,34] and V (Co_4_Ta_4_Si_7_) [34,35,36]. The G phase was first reported in 1956 by Beattie [37] in A-286 alloy. It is a kind of ternary silicide with a cubic crystal system (space group: Fm$\bar{3}$m) [38], which was named because it is easy to precipitate at the grain boundaries. Later studies found that there is a special cube-on-cube orientation relationship between the G phase and ferrite, which results in low interfacial energy, and thus makes it a potential strengthening phase of ferritic steel [39]. Additionally, the L phase was detected as a ternary MnZn_2_-type Laves phase [32]. Mittal et al. [33] design an alloy with a composition of Co_3_Ta_2_Si, which detected L and an unknown phase. In addition, the E, G″ and V phases can be treated as stoichiometric compounds and these phases are also detected in Co-Ti-Si [40] and Co-Nb-Si [41] systems. 

However, as far as we know, the phase equilibrium of the Co-Ta-Si ternary system has not been reported as far. To better understand the interaction between composition and crystal structure in Co-based superalloys, also to construct the thermodynamic database of the Co-V-Al-Ta-Ti-Ni-Cr-Si multisystem, we devote ourselves to systematically exploring the phase equilibrium relationships in ternary Co-Ta-Si alloys, a sub-system of Co-based superalloys.

**Table 1:** The stable solid phases in the Co-Ta-Si ternary systems.

System	Phase	Pearson Symbol	Prototype	Space Group	Struktur-bericht	Refs.
Co-Si	(αCo)	*cF*4	Cu	*Fm*-3*m*	A1	[26]
	(εCo)	*hP*2	Mg	*P*6_3_/*mmc*	A3	[26]
	Co_3_Si	*hP*8	Mg_3_Cd	*P*6_3_/*mmc*	-	[26]
	αCo_2_Si	*oP*12	Co_2_Si	*Pnma*	C23	[26]
	βCo_2_Si	-	-	-	-	[26]
	CoSi	*cP*8	FeSi	*P*2_1_3	B20	[26]
	CoSi_2_	*cF*12	CaF_2_	*Fm*-3*m*	C1	[26]
	(Si)	*cF*8	C(diamond)	*Fd*-3*m*	A4	[26]
Co-Ta	Co_7_Ta_2_	*hR*36	BaPb_3_	R-3m	-	[27]
	λ_1_-Co_2_Ta	*hP*12	Zn_2_Mg	*P*6_3_/*mmc*	C14	[27]
	λ_2_-Co_2_Ta	*cF*24	Cu_2_Mg	*Fd*-3*m*	C15	[27]
	λ_3_-Co_2_Ta	*hP*24	MgNi_2_	*P*6_3_/*mmc*	C36	[27]
	Co_6_Ta_7_	*hR*13	Fe_7_W_6_	*R*-3*m*	D8_5_	[27]
	CoTa_2_	*tI*12	Al_2_Cu	*I*4*/mcm*	C16	[27]
	(Ta)	*cI*2	W	*Im*-3*m*	A2	[27]
Ta-Si	Ta_3_Si	*tP*32	Ti_3_P	*P*6_3_/*mcm*	-	[28]
	Ta_2_Si	*tI1*2	Al_2_Cu	*I*4/*mcm*	C16	[28]
	αTa_5_Si_3_	*tI*32	Cr_5_B_3_	*I*4/*mcm*	D8_1_	[28]
	βTa_5_Si_3_	*hP*16	Mn_5_Si_3_	*P*6_3_/*mcm*	D8_8_	[28]
	γTa_5_Si_3_	*tI*32	W_5_Si_3_	*I*4/*mcm*	D8_m_	[28]
	TaSi_2_	*hP*9	CrSi_2_	*P*6_2_22	C40	[28]
	(Si)	*cF*8	C(diamond)	*Fd*-3*m*	A4	[28]
Co-Ta-Si	CoTaSi (E)	*oP*12	TiNiSi	*Pnma*	C23	[34]
	Co_16_Ta_6_Si_7_ (G)	*cF*116	Mg_6_Cu_16_Si_7_	*Fm*3*m*	A1	[29]
	Co_4_TaSi_3_ (G″)	*hP*168	Y_13_Pd_40_Sn_31_	*P*6*/mmm*	-	[30]
	Co_3_Ta_2_Si (L)	*hP*12	MgZn_2_	*P*6_3_/*mmc*	C14	[32]
	Co_4_Ta_4_Si_7_ (V)	*tI*60	Zr_4_Co_4_Ge_7_	-	-	[34]

## 2. Experimental Procedures

High-purity cobalt (>99.9 wt.%, bulk, Beijing Trillion Metals Co., Ltd., Beijing, China), tantalum (>99.9 wt.%, flake, Beijing Trillion Metals Co., Ltd., Beijing, China) and silicon (>99.9 wt.%, block, Beijing Trillion Metals Co., Ltd., Beijing, China) were adopted as starting materials. The samples were prepared by arc-melting using a water-cooled copper crucible with a non-consumable tungsten electrode under the high purity Ar atmosphere (DHL-1250, Sky Technology Development Co, Ltd., Shenyang, China). The weight of each sample was about 20 g and they were arc-melted at least five times to achieve the compositional uniformity. The overall weight loss after arc-melting was no more than 0.5 wt.%. Then, these samples were sealed in capsules via backfilling with Ar and annealed at 900 °C and 1100 °C, respectively. Considering the high melting point for the elemental Ta, the time of annealing was set as 90 days for 900 °C and 60 days for 1100 °C, respectively. To prevent contamination of samples, the capsules were inserted with Ti scrap and the samples were wrapped with a Ta sheet. After reaching the preset annealing time, the samples were quenched into ice water and prepared by standard metallographic methods.

The equilibrium composition of each phase was investigated by EPMA (electron probe microanalyzer) (JAX-8100R, JEOL, Tokyo, Japan) with WDS (wavelength dispersive X-ray spectroscopy) and BSE (backscattered electrons). Crystal structure analysis was carried out through XRD (X-ray diffractometer) (D8 Advance, Bruker, Karlsruhe, Germany) using Cu Kα radiation at 40.0 kV and 40 mA, and the data were collected in the range of 2θ from 10° to 90° at a step size of 0.0167°. 

## 3. Results and Discussion

### 3.1. Microstructure and Phase Equilibrium

In Figure 1, the Co-Ta-Si ternary system is divided into four regions (zone 1–4), phase equilibrium from each region is carefully discussed below. The following composition of each phase and alloy is described by the atomic ratio (at.%). The representative micrograph images and corresponding XRD indexing results are given below to indicate the phase equilibria relationship of the alloy. The nominal composition, annealing time and equilibrium composition of each phase measured by WDS are all listed in Table 2 and Table 3.

#### 3.1.1. Equilibria at Zone 1

**Figure 2:**
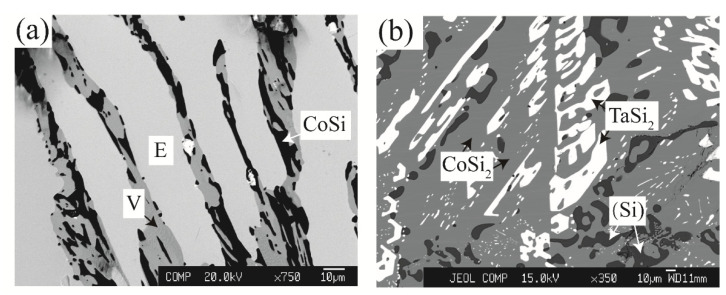
Typical ternary micrograph images obtained of (**a**) Co_34_Ta_26_Si_40_ alloy annealed at 1100 °C for 60 days and (**b**) Co_26_Ta_7_Si_67_ alloy annealed at 1100 °C for 60 days.

**Figure 3:**
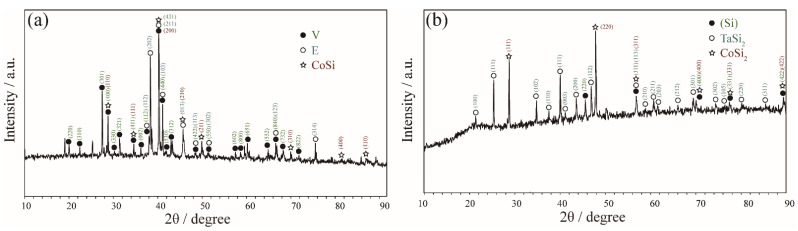
XRD patterns obtained of (**a**) Co_34_Ta_26_Si_40_ alloy annealed at 1100 °C for 60 days and (**b**) Co_26_Ta_7_Si_67_ alloy annealed at 1100 °C for 60 days.

Zone 1 mainly contains the phase equilibria at the Si-rich corner (Si > 50 at.%). For the Co_34_Ta_26_Si_40_ alloy that was annealed at 1100 °C, a distinct three-phase equilibrium of E + V + CoSi was observed in Figure 2a and their crystal structures could be confirmed by the corresponding XRD pattern in Figure 3a. As shown in Figure 2b, the Co_26_Ta_7_Si_67_ alloy is located in a three-phase equilibria region after being annealed at 1100 °C. The white and light gray phases correspond to the TaSi_2_ and CoSi_2_ phases, respectively, while the dark gray phase is presumed to be the solid solution phase of Si (signed as (Si)). The corresponding XRD pattern indexing result in Figure 3b further confirmed this speculation.

#### 3.1.2. Equilibria at Zone 2

**Figure 4:**
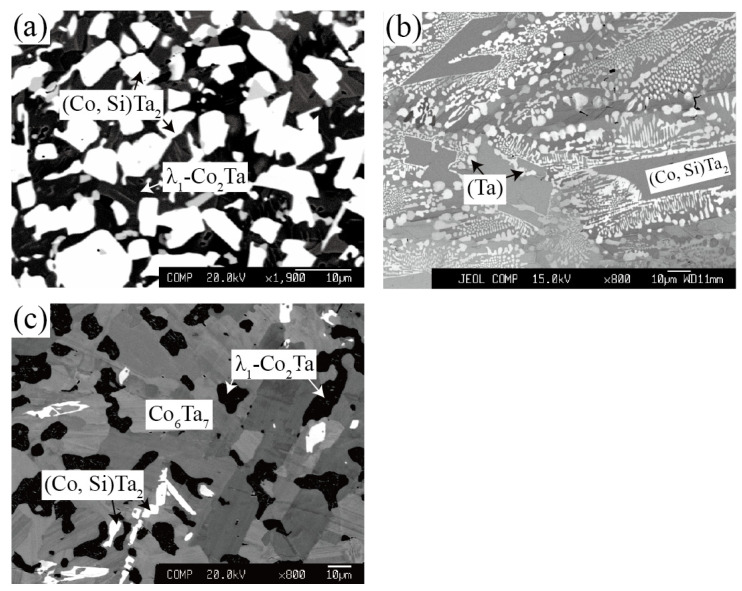
Typical ternary micrograph images obtained of (**a**) Co_30_Ta_43_Si_27_ alloy annealed at 1100 °C for 60 days; (**b**) Co_11_Ta_75_Si_14_ alloy annealed at 900 °C for 90 days and (**c**) Co_41_Ta_47_Si_12_ alloy annealed at 900 °C for 90 days.

**Figure 5:**
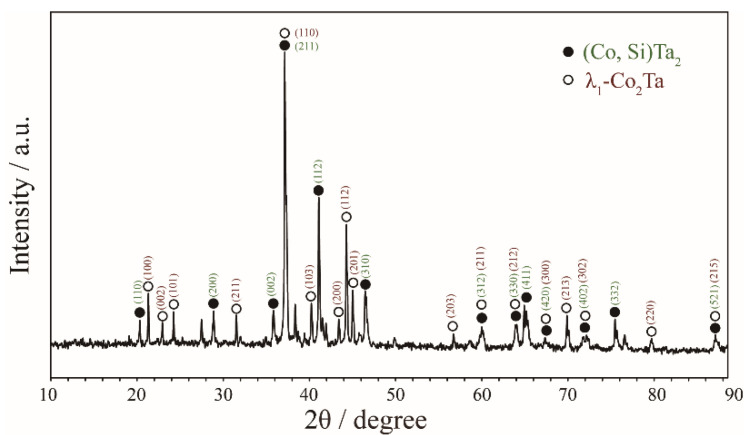
XRD patterns obtained of Co_30_Ta_43_Si_27_ alloy annealed at 1100 °C for 60 days.

Zone 2 corresponds to the phase equilibria at the Ta-rich corner (Ta > 50 at.%). In Figure 4a, a two-phase equilibrium of (Co, Si)Ta_2_ (white) + λ_1_-Co_2_Ta (black) was confirmed in the Co_30_Ta_43_Si_27_ alloy quenching from 1100 °C based on the support of the corresponding X-ray diffraction pattern in Figure 5. As shown in Figure 4b, the Co_11_Ta_75_Si_14_ alloy formed a two-phase equilibrium microstructure of (Ta) + (Co, Si)Ta_2_ after being annealed at 900 °C. Figure 4c depicts the three-phase equilibrium of (Co, Si)Ta_2_ (white) + Co_6_Ta_7_ (grey) + λ_1_-Co_2_Ta (black) in Co_41_Ta_47_Si_12_ alloy after being annealed at 900 °C.

Compositional analysis of the Co_30_Ta_43_Si_27_, Co_27_Ta_60_Si_13_, Co_36_Ta_54_Si_10_, Co_2_Ta_75_Si_23_, Co_11_Ta_75_Si_14_, Co_38_Ta_58_Si_4_, Co_10_Ta_59_Si_31_, Co_37_Ta_42_Si_21_, Co_41_Ta_47_Si_12_, Co_27_Ta_68_Si_5_ and Co_26_Ta_53_Si_21_ alloys indicate that these alloys contain a phase with about 63 at.% Ta after being annealed at 900 °C and 1100 °C. The X-ray diffraction analysis results of these alloys strongly suggest that the binary Al_2_Cu-type CoTa_2_ and Ta_2_Si phases form a continuous solid solution phase (Co, Si)Ta_2_. As far as we know, it is the first time to discover the infinite mutual solubility between CoTa_2_ and Ta_2_Si phases. Similarly, the phenomenon of Ni and Si can be entirely substituted by each other in Al_2_Cu-type compounds was also found in the Ni-Ta-Si ternary system [42].

#### 3.1.3. Equilibria at Zone 3

**Figure 6:**
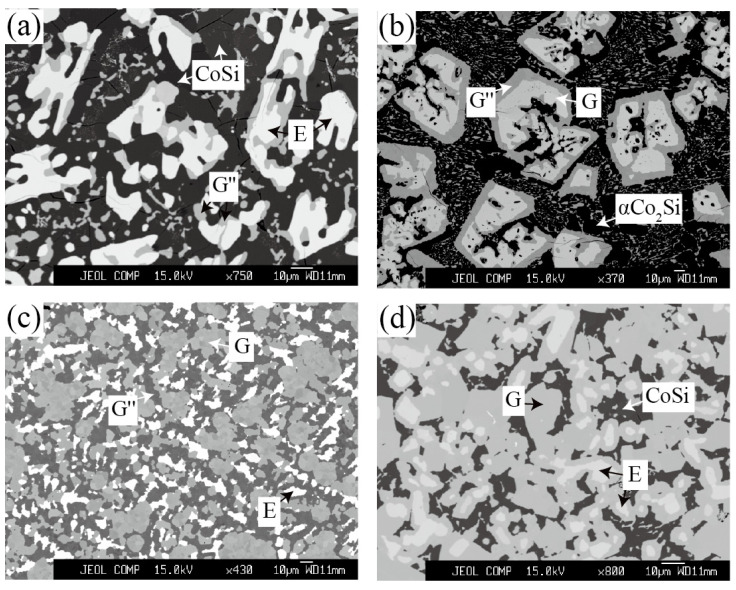
Typical ternary micrograph images obtained of (**a**) Co_46_Ta_13_Si_41_ alloy annealed at 1100 °C for 60 days; (**b**) Co_58_Ta_10_Si_32_ alloy annealed at 1100 °C for 60 days; (**c**) Co_48_Ta_22_Si_30_ alloy annealed at 1100 °C for 60 days and (**d**) Co_48_Ta_22_Si_30_ alloy annealed at 900 °C for 90 days.

**Figure 7:**
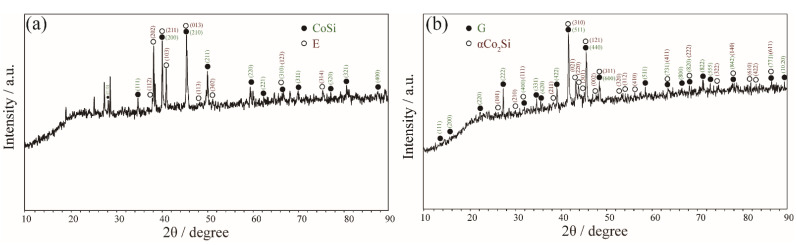
XRD patterns obtained of (**a**) Co_46_Ta_13_Si_41_ alloy annealed at 1100 °C for 60 days and (**b**) Co_58_Ta_10_Si_32_ alloy annealed at 1100 °C for 60 days.

Zone 3 mainly discusses the phase equilibria around G″ phase. The alloy Co_46_Ta_13_Si_41_, Co_58_Ta_10_Si_32_ and Co_48_Ta_22_Si_30_ were designed to explore the existence of the G″ phase. For Co_46_Ta_13_Si_41_ and Co_58_Ta_10_Si_32_ alloys that being annealed at 1100 °C, two three-phase equilibrium CoSi + E + G″ and αCo_2_Si + G + G″ were detected as shown in Figure 6a,b. The corresponding XRD patterns in Figure 7a,b indicate that the diffraction peaks belonging to the phases (CoSi, E, αCo_2_Si and G) are in good agreement with the standard patterns. However, the diffraction peak of the G″ phase was not interpreted due to the lack of crystallographic information. For the Co_48_Ta_22_Si_30_ alloy annealed at 1100 °C, a clearly three-phase equilibria E (white) + G (light gray) + G″ (dark gray) was detected, as shown in Figure 6c. However, the EMPA micrographs and WDS analysis of the same alloy (see Figure 6d) quenching from 900 °C show that it is a three-phase of E + G + CoSi. This result implies that the G″ phase is a high-temperature compound, which is not stable at 900 °C.

#### 3.1.4. Equilibria at Zone 4

**Figure 8:**
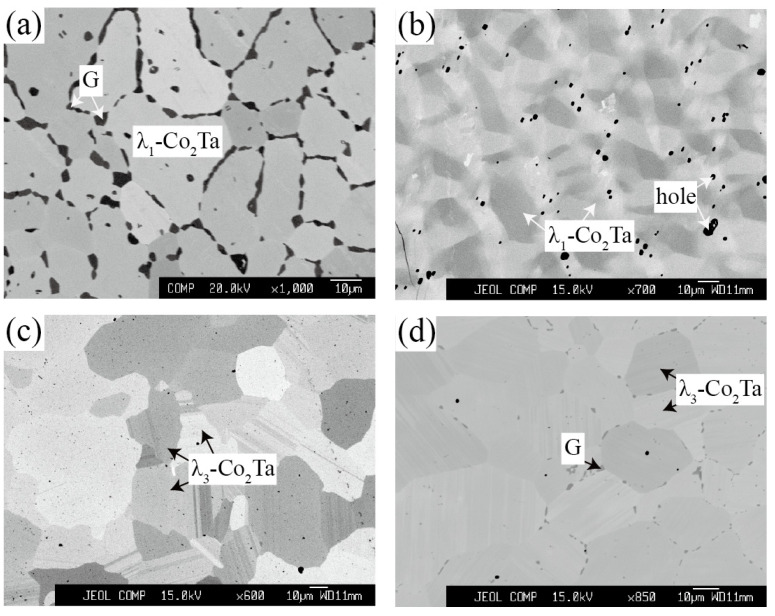
Typical ternary micrograph images obtained of (**a**) Co_54_Ta_28_Si_18_ alloy annealed at 1100 °C for 60 days; (**b**) Co_59_Ta_31_Si_10_ alloy annealed at 1100 °C for 60 days; (**c**) Co_65_Ta_27_Si_8_ alloy annealed at 1100 °C for 60 days and (**d**) Co_65_Ta_27_Si_8_ alloy annealed at 900 °C for 90 days.

Zone 4 describes the phase equilibria of other ternary phases including G and L. As shown in Figure 8a, the dark grey G phase was observed at the grain boundaries of the light grey λ_1_-Co_2_Ta phase for the Co_54_Ta_28_Si_18_ alloy after being annealed at 1100 °C. This morphology is extremely similar to that of the G phase precipitation on the C14 Laves phase [42]. 

**Figure 9:**
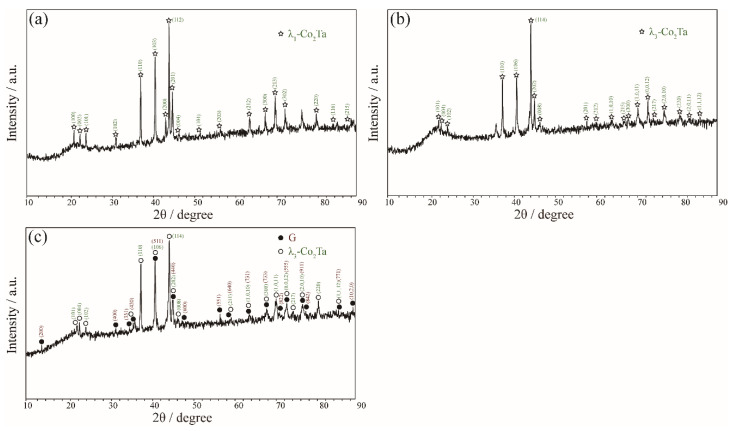
XRD patterns obtained of (**a**) Co_59_Ta_31_Si_10_ alloy annealed at 1100 °C for 60 days; (**b**) Co_65_Ta_27_Si_8_ alloy annealed at 1100 °C for 60 days and (**c**) Co_65_Ta_27_Si_8_ alloy annealed at 900 °C for 90 days.

The alloy with a nominal composition of Co_59_Ta_31_Si_10_ was designed to detect the L phase as shown in Figure 8b. The interpretation of the corresponding XRD pattern in Figure 9a indicates that this ternary L phase crystalizes the same as the Zn_2_Mg-type compounds (P6_3_/*mmc*, C14) like the binary λ_1_-Co_2_Ta phase. Additionally, the λ_1_-Co_2_Ta phase is a high-temperature phase that only exists above 1294 °C [27]. However, the detected ternary L phase is stable at 900 °C and 1100 °C, suggesting that the addition of Si stabilizes the λ_1_-Co_2_Ta phase toward low temperature. Besides, this Co-Ta-Si ternary L phase shows an extremely similar compositional range compared with the Ni-Nb-Si system [43]. The alloy Co_65_Ta_27_Si_8_ was designed to determine the phase equilibria of the G and three Laves phases (λ_1_-Co_2_Ta, λ_2_-Co_2_Ta and λ_3_-Co_2_Ta). A single λ_3_-Co_2_Ta phase was detected in Co_65_Ta_27_Si_8_ alloy annealed at 1100 °C (Figure 8c), while the λ_3_-Co_2_Ta + G was observed for the same alloy being annealed at 900 °C (Figure 8d). The corresponding XRD patterns in Figure 9b,c confirm the above equilibrium relationship.

### 3.2. Isothermal Sections

**Figure 10:**
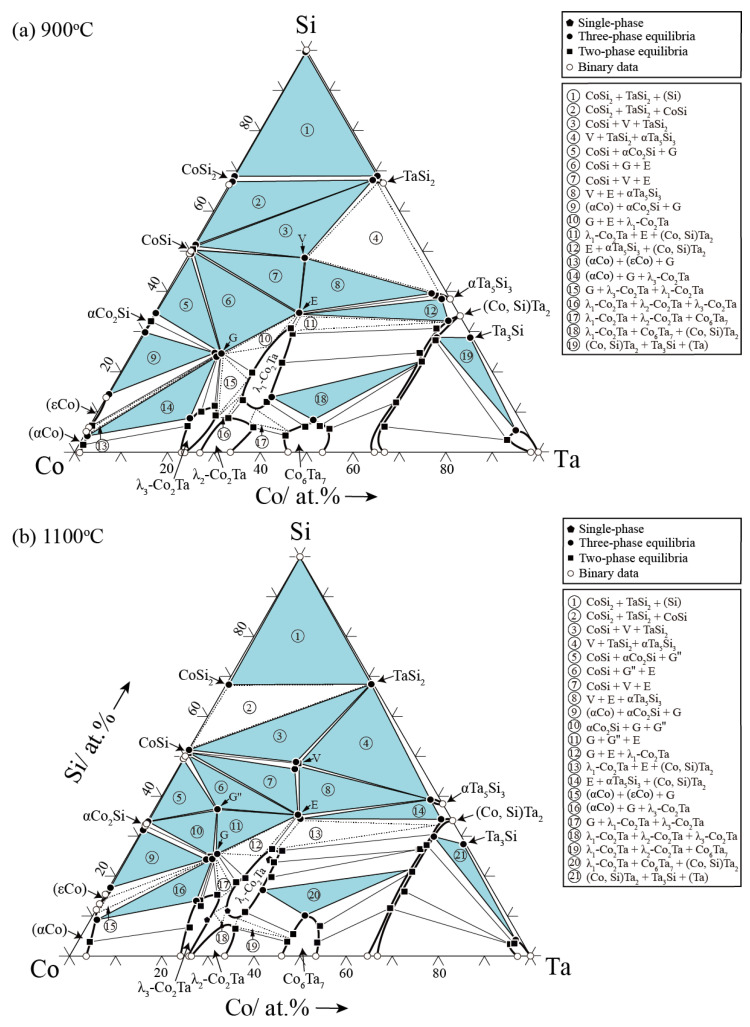
Experimental determined isothermal sections of the Co-Ta-Si system at (**a**) 900 °C and (**b**) 1100 °C.

Based on the phase equilibrium information discussed in Section 3.1, the isothermal sections of the Co-Ta-Si ternary system at 900 °C and 1100 °C are constructed in the whole composition range (see Figure 10). Nineteen and twenty-one three-phase regions were observed at 900 °C and 1100 °C, respectively. Few undetected three-phase regions are indicated by the dashed line.

The solid solubilities of Ta in the Co-Si binary phases (CoSi_2_, CoSi and αCo_2_Si) are negligible. The maximum solubility of Co in the TaSi_2_ phase is 1.7 at.% at 1100 °C, and it increases to 3.4 at.% at 900 °C. When the temperature decreases from 1100 °C to 900 °C, the solid solubility of Co in the αTa_5_Si_3_ phase increases from 3.5 at.% to 5.3 at.%. In addition, the maximum solubility of Si in the λ_3_-Co_2_Ta phase was measured to be 15.3 at.% at 1100 °C and decreased to 11.5 at.% at 900 °C. At least 8.5 at.% of Si can be dissolved in the λ_2_-Co_2_Ta phase at 900 °C. However, the solid solubility of Si in Co_6_Ta_7_ hardly varies with temperature, maintaining 12 at.% at both 900 °C and 1100 °C.

In the Co-Ta-Si ternary system, there are four ternary compound phases G, G″, E and V and a binary high-temperature phase λ_1_-Co_2_Ta (L) stabilized by Si. The G, E, V and G″ phases are stoichiometric compounds exhibiting only small homogeneity ranges. Meanwhile, the G, E, V and L phases exist at 900 °C and 1100 °C. The G″ phase has also been reported in similar ternary systems like the Co-Nb-Si [41] and Co-Ti-Si [40]. The G″ phase was detected in the Co_46_Ta_13_Si_41_, Co_58_Ta_10_Si_32_ and Co_48_Ta_22_Si_30_ alloys after being annealed at 1100 °C, but it disappears at 900 °C.

## 4. Conclusions

The phase equilibrium of the Co-Ta-Si ternary system at 900 °C and 1100 °C is systematically studied by equilibrated alloy and the following conclusions can be drawn:

1. The four known ternary phases, G (Co_16_Ta_6_Si_7_), E (CoTaSi), G″ (Co_4_TaSi_3_) and V (Co_4_Ta_4_Si_7_) have almost no ternary solid solubilities, which could be treated as stoichiometric compounds.
2. The high-temperature phase G″ (Co_4_TaSi_3_) is only stable at 1100 °C and it disappears when the temperature decreases to 900 °C.
3. The addition of Si increases the thermal stability of the binary λ_1_-Co_2_Ta (C14 Laves) phase, resulting in the formation of the ternary L phase with the composition of Co_32.3–58.8_Ta_28.2–36.8_Si_13.0–30.9_ at 900 °C and Co_39.3–62.3_Ta_26.8–33.9_Si_10.9–26.8_ at 1100 °C.
4. Both the binary CoTa_2_ and SiTa_2_ phases crystallize with the same body-centered tetragonal structure (space group: I4/*mcm*, C16) and they form a continuous solid solution phase (Co, Si)Ta_2_.
5. The maximum solid solubility of Si for the λ_3_-Co_2_Ta phase is ~15.3 at.% at 1100 °C and it slightly decreases to be ~11.5 at.% at 900 °C. The solid solubility of Si for the Co_6_Ta_7_ phase is always ~12 at.% and does not change with temperature.
6. The elemental Ta is hardly dissolved in the CoSi_2_, CoSi and α-Co_2_Si phases. Similarly, the elemental Co has negligible solubilities in the TaSi_2_ and α-Ta_5_Si_3_ phases.

The results obtained from the present work make up the phase diagram information of the Co-Ta-Si ternary system, also provide the key experimental data for the establishment of Co-based superalloys thermodynamic database.

## Figures and Tables

**Table 2:** Equilibrium compositions of the Co-Ta-Si ternary system at 900 °C determined in the present work.

Alloy (at.%)	Annealed Time	Phase Equilibria	Composition (at.%)					
		Phase 1/Phase 2/Phase 3	Phase 1		Phase 2		Phase 3	
			Ta	Si	Ta	Si	Ta	Si
Co_10_Ta_10_Si_80_	90 days	TaSi_2_ / CoSi_2_ / (Si)	30.3	68.6	0.1	70.3	0.1	99.7
Co_33_Ta_14_Si_53_	90 days	V / CoSi	24.8	48.4	0.1	51.6		
Co_30_Ta_43_Si_27_	90 days	(Co, Si)Ta_2_ / λ_1_-Co_2_Ta	62.6	30.8	32.2	29.5		
Co_54_Ta_28_Si_18_	90 days	λ_1_-Co_2_Ta / G	28.2	18.9	19.2	25.1		
Co_16_Ta_18_Si_66_	90 days	TaSi_2_ / CoSi / CoSi_2_	30.4	67.8	0.2	51.8	0.2	67.2
Co_22_Ta_27_Si_51_	90 days	TaSi_2_ / V / CoSi	30.6	67.8	25.3	48.2	0.5	51.6
Co_27_Ta_60_Si_13_	90 days	(Co, Si)Ta_2_ / Co_6_Ta_7_	63.7	22.8	49.6	6.7		
Co_34_Ta_26_Si_40_	90 days	E / V / CoSi	31.6	34.8	22.7	46.5	0.2	50.5
Co_25_Ta_38_Si_37_	90 days	αTa_5_Si_3_ / E / V	58.4	39.0	32.1	35.2	25.2	48.1
Co_64.5_Ta_22.5_Si_13_	90 days	λ_3_-Co_2_Ta / G	23.5	11.5	19.9	25.4		
Co_36_Ta_54_Si_10_	90 days	(Co, Si)Ta_2_ / Co_6_Ta_7_	64.0	20.6	49.7	9.2		
Co_2_Ta_75_Si_23_	90 days	(Ta) / (Co, Si)Ta_2_ / Ta_3_Si	92.3	5.4	63.8	28.6	71.0	28.6
Co_26_Ta_7_Si_67_	90 days	CoSi_2_ / TaSi_2_	0.1	67.5	29.5	67.1		
Co_46_Ta_13_Si_41_	90 days	G / CoSi	18.2	25.8	0.1	51.2		
Co_58_Ta_10_Si_32_	90 days	Co_2_Si / CoSi / G	0.1	34.6	0.1	50.6	18.0	26.2
Co_48_Ta_22_Si_30_	90 days	E / G / CoSi	30.2	35.1	18.3	26.1	0.1	51.1
Co_36_Ta_33_Si_31_	90 days	E / λ_1_-Co_2_Ta	30.9	35.2	31.1	30.9		
Co_59.5_Ta_22.5_Si_18_	90 days	λ_3_-Co_2_Ta / G	22.1	11.0	18.6	24.6		
Co_59_Ta_31_Si_10_	90 days	λ_1_-Co_2_Ta / λ_2_-Co_2_Ta	29.7	13.0	28.8	8.5		
Co_11_Ta_75_Si_14_	90 days	(Ta) / (Co, Si)Ta_2_	91.2	3.0	63.7	28.1		
Co_38_Ta_58_Si_4_	90 days	Co_6_Ta_7_ / (Co, Si)Ta_2_	63.3	12.4	52.1	5.9		
Co_25_Ta_33_Si_42_	90 days	αTa_5_Si_3_ / E / V	57.2	39.6	32.4	35.6	24.4	48.9
Co_24_Ta_44_Si_31_	90 days	αTa_5_Si_3_ / E	58.1	39.4	31.6	34.8		
Co_65_Ta_27_Si_8_	90 days	G / λ_3_-Co_2_Ta	19.1	24.1	25.3	9.2		
Co_56_Ta_9_Si_35_	90 days	G / αCo_2_Si / CoSi	19.0	25.0	0.4	33.6	0.3	49.2
Co_44_Ta_25_Si_31_	90 days	E / G / CoSi	30.7	33.6	19.3	24.6	0.7	47.4
Co_10_Ta_59_Si_31_	90 days	αTa_5_Si_3_ / E / (Co, Si)Ta_2_	58.8	39.4	32.0	29.7	63.4	33.2
Co_61_Ta_11_Si_28_	90 days	G / αCo_2_Si	17.7	24.8	0.1	32.6		
Co_70_Ta_9_Si_21_	90 days	G / (εCo) / αCo_2_Si	57.7	24.3	0.1	14.4	0.2	29.7
Co_37_Ta_42_Si_21_	90 days	(Co, Si)Ta_2_ / λ_1_-Co_2_Ta	62.0	24.7	44.3	22.2		
Co_71_Ta_13_Si_16_	90 days	G / (αCo)	18.8	24.4	0.5	6.7		
Co_78_Ta_10_Si_12_	90 days	λ_3_-Co_2_Ta / G / (αCo)	20.6	8.5	18.7	23.7	0.7	4.0
Co_51_Ta_37_Si_12_	90 days	Co_6_Ta_7_ / λ_1_-Co_2_Ta	46.5	6.2	32.4	16.2		
Co_41_Ta_47_Si_12_	90 days	(Co, Si)Ta_2_ / Co_6_Ta_7_ / λ_1_-Co_2_Ta	62.9	22.3	47.6	8.9	36.8	13.5
Co_78_Ta_17_Si_5_	90 days	λ_3_-Co_2_Ta / (αCo)	21.0	6.4	0.9	1.9		
Co_57_Ta_38_Si_5_	90 days	Co_6_Ta_7_ / λ_2_-Co_2_Ta	43.2	4.1	35.5	6.1		
Co_27_Ta_68_Si_5_	90 days	(Ta) / (Co, Si)Ta_2_	91.7	2.9	63.5	6.2		

**Table 3:** Equilibrium compositions of the Co-Ta-Si ternary system at 1100 °C determined in the present work.

Alloy (at.%)	Annealed Time	Phase Equilibria	Composition (at.%)					
		Phase 1/Phase 2/Phase 3	Phase 1		Phase 2		Phase 3	
			Ta	Si	Ta	Si	Ta	Si
Co_10_Ta_10_Si_80_	60 days	TaSi_2_ / CoSi_2_ / (Si)	31.0	67.3	0.7	68.0	0.1	99.0
Co_33_Ta_14_Si_53_	60 days	V / CoSi	25.4	48.2	0.1	50.9		
Co_10_Ta_38_Si_52_	60 days	TaSi_2_ / αTa_5_Si_3_ / V	31.4	68.1	58.5	39.3	25.6	48.8
Co_46_Ta_28_Si_26_	60 days	E / G	30.8	34.8	19.7	25.5		
Co_30_Ta_43_Si_27_	60 days	(Co, Si)Ta_2_ / λ_1_-Co_2_Ta	63.6	30.3	32.9	26.4		
Co_54_Ta_28_Si_18_	60 days	λ_1_-Co_2_Ta / G	28.2	19.5	20.1	25.4		
Co_16_Ta_18_Si_66_	60 days	CoSi / TaSi_2_ / V	30.8	67.6	0.1	51.8	24.5	48.4
Co_22_Ta_27_Si_51_	60 days	TaSi_2_ / V	31.4	68.4	25.3	48.9		
Co_27_Ta_60_Si_13_	60 days	(Co, Si)Ta_2_ / Co_6_Ta_7_	63.8	21.1	50.7	7.5		
Co_35_Ta_5_Si_60_	60 days	V / CoSi	23.8	48.6	0.1	51.2		
Co_34_Ta_26_Si_40_	60 days	E / V / CoSi	31.8	35.4	25.2	46.8	0.1	51.9
Co_25_Ta_38_Si_37_	60 days	E / αTa_5_Si_3_ / V	57.7	39.6	32.0	35.6	25.7	48.3
Co_64.5_Ta_22.5_Si_13_	60 days	λ_3_-Co_2_Ta / G	22.1	12.6	19.5	23.8		
Co_2_Ta_75_Si_23_	60 days	Ta / (Co, Si)Ta_2_ / Ta_3_Si	94.8	4.0	64.0	30.0	27.9	71.6
Co_26_Ta_7_Si_67_	60 days	TaSi_2_ / CoSi_2_ / (Si)	30.4	68.1	0.1	67.8	0.1	99.5
Co_46_Ta_13_Si_41_	60 days	E / G″ / CoSi	30.2	35.0	13.0	37.4	0.1	50.5
Co_58_Ta_10_Si_32_	60 days	αCo_2_Si / G / G″	0.1	34.7	18.6	25.8	13.6	37.1
Co_48_Ta_22_Si_30_	60 days	G / G″ / E	18.7	25.9	13.4	37.3	30.7	34.8
Co_36_Ta_33_Si_31_	60 days	E / λ_1_-Co_2_Ta	30.7	35.4	30.5	26.8		
Co_59.5_Ta_22.5_Si_18_	60 days	G / λ_3_-Co_2_Ta	18.8	25.1	24.2	15.3		
Co_59_Ta_31_Si_10_	60 days	λ_1_-Co_2_Ta	28.7	11.3				
Co_11_Ta_75_Si_14_	60 days	(Ta) / (Co, Si)Ta_2_	95.0	3.1	63.3	27.9		
Co_38_Ta_58_Si_4_	60 days	Co_6_Ta_7_ / (Co, Si)Ta_2_	63.5	12.0	52.1	3.4		
Co_25_Ta_33_Si_42_	60 days	E / V	31.4	35.9	24.6	48.8		
Co_24_Ta_44_Si_31_	60 days	αTa_5_Si_3_ / E	58.6	39.4	30.8	35.5		
Co_65_Ta_27_Si_8_	60 days	λ_3_-Co_2_Ta	25.1	9.1				
Co_42_Ta_15_Si_43_	60 days	CoSi / E	0.3	50.8	30.0	35.0		
Co_26_Ta_33_Si_41_	60 days	V / E	24.9	48.5	31.3	35.1		
Co_56_Ta_9_Si_35_	60 days	G" / αCo_2_Si / CoSi	13.6	36.5	0.1	33.9	0.1	49.3
Co_44_Ta_25_Si_31_	60 days	G / G"	18.9	25.6	13.9	36.4		
Co_10_Ta_59_Si_31_	60 days	αTa_5_Si_3_ / Ta_2_Si / E	58.7	39.1	63.4	34.1	32.9	34.4
Co_61_Ta_11_Si_28_	60 days	G / αCo_2_Si	18.1	24.9	0.2	32.7		
Co_70_Ta_9_Si_21_	60 days	G / (εCo) / αCo_2_Si	17.9	24.6	0.2	17.4	0.1	32.0
Co_54_Ta_25_Si_21_	60 days	λ_1_-Co_2_Ta / G	26.8	18.5	19.2	25.6		
Co_54_Ta_25_Si_21_	60 days	λ_1_-Co_2_Ta	31.2	24.1				
Co_37_Ta_42_Si_21_	60 days	(Co, Si)Ta_2_ / λ_1_-Co_2_Ta	62.4	26.6	32.5	23.5		
Co_26_Ta_53_Si_21_	60 days	(Co, Si)Ta_2_ / λ_1_-Co_2_Ta	62.7	26.9	33.9	19.9		
Co_60_Ta_24_Si_16_	60 days	λ_3_-Co_2_Ta / G	21.3	13.9	18.9	24.5		
Co_78_Ta_10_Si_12_	60 days	λ_3_-Co_2_Ta / G / (αCo)	20.6	13.8	18.8	24.2	1.3	8.9
Co_51_Ta_37_Si_12_	60 days	Co_6_Ta_7_ / λ_1_-Co_2_Ta	45.8	5.5	32.6	10.9		
Co_41_Ta_47_Si_12_	60 days	(Co, Si)Ta_2_ / Co_6_Ta_7_ / λ_1_-Co_2_Ta	62.6	21.5	45.9	10.1	33.6	16.5
Co_21_Ta_67_Si_12_	60 days	(Co, Si)Ta_2_ / (Ta)	94.1	3.0	63.6	24.5		
Co_78_Ta_17_Si_5_	60 days	λ_3_-Co_2_Ta / (αCo)	22.4	7.1	2.6	3.5		
Co_57_Ta_38_Si_5_	60 days	Co_6_Ta_7_ / λ_2_-Co_2_Ta	45.3	3.6	32.5	6.8		

## Data Availability

Data sharing not applicable.
